# Reference genome for the endangered, genetically subdivided, northern tidewater goby, *Eucyclogobius newberryi*

**DOI:** 10.1093/jhered/esae053

**Published:** 2024-10-05

**Authors:** David K Jacobs, Andrew Kinziger, Mira Abrecht, W Tyler McCraney, Benjamin A Hà, Brenton T Spies, Elizabeth Heath-Heckman, Mohan P A Marimuhtu, Oanh Nguyen, Colin W Fairbairn, William E Seligmann, Merly Escalona, Courtney Miller, H Bradley Shaffer

**Affiliations:** Department of Ecology and Evolutionary Biology, University of California, Los Angeles, California, USA; Department of Fisheries Biology, Cal Poly Humboldt, Arcata, California, USA; Department of Ecology and Evolutionary Biology, University of California, Los Angeles, California, USA; Department of Fisheries Biology, Cal Poly Humboldt, Arcata, California, USA; Department of Ecology and Evolutionary Biology, University of California, Los Angeles, California, USA; Environmental Science and Resource Management Program, California State University Channel Islands, Camarillo, California, USA; Department of Integrative Biology, Michigan State University, East Lansing, Michigan, USA; Department of Microbiology, Genetics, and Immunology, Michigan State University, East Lansing, Michigan, USA; DNA Technologies and Expression Analysis Core Laboratory, Genome Center, University of California, Davis, California, USA; DNA Technologies and Expression Analysis Core Laboratory, Genome Center, University of California, Davis, California, USA; Department of Ecology and Evolutionary Biology, University of California, Santa Cruz, California, USA; Department of Ecology and Evolutionary Biology, University of California, Santa Cruz, California, USA; Department of Biomolecular Engineering, University of California Santa Cruz, California, USA; Department of Ecology and Evolutionary Biology, University of California, Los Angeles, California, USA; La Kretz Center for California Conservation Science, Institute of the Environment and Sustainability, University of California, Los Angeles, Los Angeles, California, USA; Department of Ecology and Evolutionary Biology, University of California, Los Angeles, California, USA; La Kretz Center for California Conservation Science, Institute of the Environment and Sustainability, University of California, Los Angeles, Los Angeles, California, USA

**Keywords:** California Conservation Genomics Project, CCGP, conservation genetics, genomics, Gobiidae, metapopulation

## Abstract

The federally endangered sister species, *Eucyclogobius newberryi* (northern tidewater goby, NTG) and *E. kristinae* (southern tidewater goby) comprise the California endemic genus *Eucyclogobius*, which historically occurred in all coastal California counties. Isolated lagoons that only intermittently connect to the sea are their primary habitat. Reproduction occurs during lagoon closure, minimizing marine dispersal and generating the most genetically subdivided vertebrate genus on the California coast. We present a new genome assembly for *E. newberryi* using HiFi long reads and Hi-C chromatin-proximity sequencing. The 980 Mb *E. newberryi* reference genome has an N50 of 34 Mb with 22 well-described scaffolds comprising 88% of the genome and a complete BUSCO (Benchmarking Universal Single-Copy Orthologs) score of 96.7%. This genome will facilitate studies addressing selection, drift, and metapopulation genetics in subdivided populations, as well as the persistence of the critically endangered *E. kristinae*, where reintroduction will be an essential element of conservation actions for recovery. It also provides tools critical to the recovery of the genetically distinct management units in the NTG, as well as broader ecological and evolutionary studies of gobies, the most speciose family of fishes in the world.

## Introduction

Tidewater gobies (TWG) of the genus *Eucyclogobius* occur only in California where they primarily inhabit coastal lagoons that are often isolated from the sea and exhibit high levels of regional genetic distinction and local genetic subdivision between lagoons (Swift et al. 1989; Dawson et al. 2001; Earl et al. 2010). These lagoons vary in hydrology, temperature, disease prevalence, and other environmental factors (Jacobs et al. 2011; Spies and Steele 2016; Feyrer et al. 2023), creating the potential for extreme local adaptation (Ahnelt et al. 2004; Chase et al. 2016; Swift et al. 2016). Many lagoons are also limited in size, supporting small, annually variable TWG populations that are subject to extensive genetic drift (USFWS 2005, 2013; McCraney et al. 2010; Spies 2022). Rainfall events intermittently open TWG lagoonal habitat to the sea, allowing episodic marine dispersal over modest distances (Jacobs et al. 2004; Ha 2023), while desiccation in extended drought eliminates many habitats which then recolonizes during wetter periods. These factors yield varied local and regional metapopulation processes, with episodic extinction and colonization of individual sites (Lafferty et al. 1999; Kinziger et al. 2015; Martel et al. 2021).

The genus *Eucyclogobius* was long considered monotypic, and its sole species *E. newberryi* (Girard 1856) was listed as federally endangered under the Endangered Species Act in 1994 due to a reduction in the number of occupied sites over the previous decades (USFWS 2005). However, *E. newberryi* was recently split into northern and southern species based on genetic divergence and morphologic character differences (Swift et al. 2016). The name *E. newberryi* was retained for the northern species now designated as the northern tidewater goby (NTG), which ranges from northern Los Angeles County to Del Norte County near the California–Oregon border. The NTG is managed by the USFWS in five recovery units (Dawson et al. 2001; USFWS 2005, 2013; Earl et al. 2010; McCraney et al. 2010; Kinziger et al. 2015). *Eucyclogobius kristinae*, the southern tidewater goby (STG), is currently restricted to fewer than five lagoons on Camp Pendleton Marine Base in northern San Diego County (Swift et al. 2016; Spies 2022).

Here we present a reference genome for the NTG, one of approximately 250 species included in the California Conservation Genomics Project (CCGP: Shaffer et al. 2022). The genome assembly fills an important phylogenetic gap in our coverage of California reference genomes (Toffelmier et al. 2022), and will facilitate population genomics inference and improve the effectiveness of management and conservation strategies for endangered *Eucyclogobius* species (Fiedler et al. 2022).

## Methods

### Biological materials

**Fig. 1.**
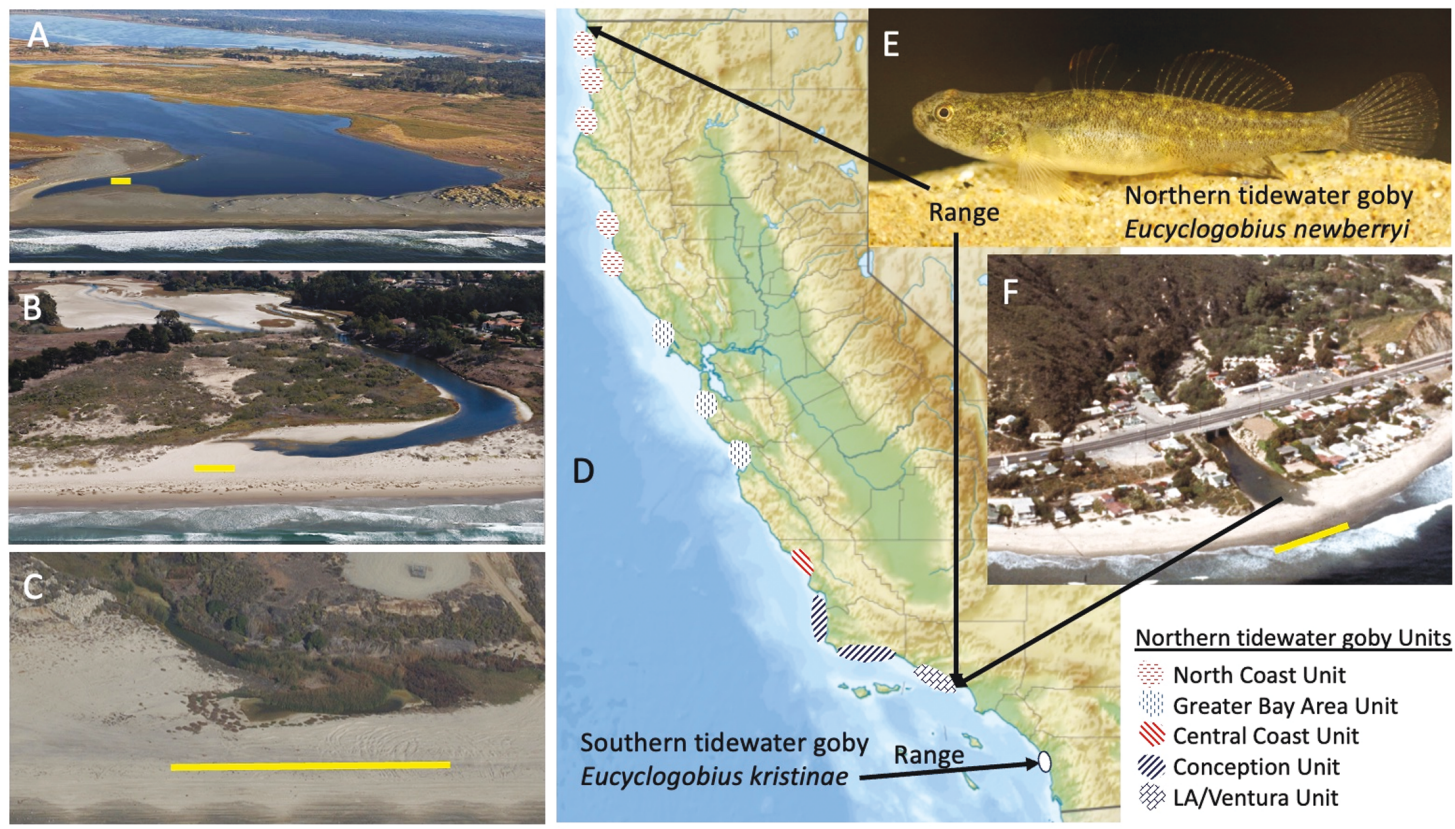
Tidewater goby range and habitat. Panels A through C and F illustrate the variation in size and degree of filling/desiccation of tidewater goby closing lagoon habitat. The scale bar represents 100 m in all cases. All coastal images are courtesy of Kenneth & Gabrielle Adelman/California Coastal Protection Network. A) Lake Earl/Talawa, a large lagoonal *E. newberryi* (NTG) habitat in Northern California. B) Devereux Lagoon, a frequently extirpated NTG site west of Santa Barbara is shown in a partially desiccated state. C) Cockleburr Lagoon is a small highly vegetated *E. kristinae* (STG) site in northern San Diego County. D) The large range of the NTG contrasts with the dramatically reduced range of the STG. Patterned dots in the key mark the general location of habitat concentrations within the North Coast, Greater Bay Area, Central Coast, Point Conception, and LA/Ventura management units of the NTG. E) NTG from Carpenter Creek, San Luis Obispo County. F) Topanga Lagoon in 1972, where channelization, bridging, and coastal development have significantly impacted the lagoon as is typical of many current and former tidewater goby sites in Southern California and the San Francisco Bay Area. Topanga Lagoon is the source of the individual/DNA used in our reference genome construction.

One adult NTG, *E. newberryi* (about 50 to 60 mm total length) was collected by seine at Topanga Lagoon, Los Angeles County, California (34.03839 N to 118.5831 W) on 17 June 2021 by Dave Jacobs, Mira Abrecht, and James Brown under United States Fish and Wildlife federal recovery sub permit FWSVFWO-24.9 (Fig. 1). The fish was dissected in the field, and fin and muscle tissues were immediately placed on dry ice until transferred to a −80 °C freezer.

### High-molecular-weight genomic DNA isolation

High-molecular-weight (HMW) genomic DNA (gDNA) was extracted from 16 mg of caudal fin tissue using the Nanobind Tissue Big DNA kit (Pacific BioSciences—PacBio, Menlo Park, CA) following the manufacturer’s instructions. We assessed DNA purity using absorbance ratios (260/280 = 1.95 and 260/230 = 3.29) measured using the NanoDrop ND-1000 spectrophotometer. The DNA yield (7.2 µg) was quantified using a Quantus Fluorometer (QuantiFluor ONE dsDNA Dye assay; Promega, Madison, WI). The size distribution of the resulting HMW DNA was assessed using the Femto Pulse system (Agilent, Santa Clara, CA), which revealed that 42% of the fragments were 25 kb or longer.

### HiFi library preparation and sequencing

The HiFi SMRTbell library was constructed using the SMRTbell Express Template Prep Kit v2.0 (PacBio, Cat. #100-938-900) according to the manufacturer’s instructions. HMW gDNA was sheared to a target DNA size distribution between 15 and 18 kb using Diagenode’s Megaruptor 3 system (Diagenode, Belgium; Cat. B06010001). The sheared gDNA was concentrated using 0.45× of AMPure PB beads (PacBio, Cat. #100-265-900) for the removal of single-strand overhangs at 37 °C for 15 min, followed by further enzymatic steps of DNA damage repair at 37 °C for 30 min, end repair and A-tailing at 20 °C for 10 min and 65 °C for 30 min, and ligation of overhang adapters v3 at 20 °C for 60 min. The SMRTbell library was purified and concentrated with 1× Ampure PB beads for nuclease treatment at 37 °C for 30 min, followed by size selection using the BluePippin/PippinHT system (Sage Science, Beverly, MA; Cat #BLF7510/HPE7510) to collect fragments greater than 7 to 9 kb. The 15 to 20 kb average HiFi SMRTbell library was sequenced at UC Davis DNA Technologies Core (Davis, CA) using two 8M SMRT cells, Sequel II sequencing chemistry 2.0, and 30-h movies each on a PacBio Sequel II sequencer.

### Omni-C library preparation and sequencing

The Omni-C library was prepared using the Dovetail Omni-C Kit (Dovetail Genomics, CA) according to the manufacturer’s protocol with slight modifications. First, specimen tissue was thoroughly ground with a mortar and pestle while cooled with liquid nitrogen. Subsequently, the cell pellet was treated with 0.3 M DSG (incubated for 10 min) followed by 37% formaldehyde incubation (again for 10 min). These together crosslink the chromatin, thereby fixing it in place in the nucleus. The suspended chromatin solution was then passed through 100 and 40 µm cell strainers to remove large debris. Fixed chromatin was digested under various conditions of DNase I until a suitable fragment length distribution of DNA molecules was obtained. Chromatin ends were repaired and ligated to a biotinylated bridge adapter followed by proximity ligation of adapter-containing ends. After proximity ligation, crosslinks were reversed, and the DNA was purified from proteins. Purified DNA was treated to remove biotin that was not internal to ligated fragments. An NGS library was generated using an NEB Ultra II DNA Library Prep kit (NEB, Ipswich, MA) with an Illumina-compatible y-adaptor. Biotin-containing fragments were then captured using streptavidin beads. The post-capture product was split into two replicates prior to PCR (Polymerase Chain Reaction) enrichment to preserve library complexity with each replicate receiving unique dual indices. The library was sequenced at the Vincent J. Coates Genomics Sequencing Lab (Berkeley, CA) on an Illumina NovaSeq 6000 platform (Illumina, CA) to generate approximately 100 million 2 × 150 bp read pairs per GB genome size.

### Nuclear genome assembly

We assembled the genome of the NTG following the CCGP assembly pipeline Version 5.0 as outlined in Table 1, which lists the tools and non-default parameters used in the assembly. The pipeline uses PacBio HiFi reads and Omni-C data to produce high-quality and highly contiguous genome assemblies. First, we removed the remnants adapter sequences from the PacBio HiFi dataset using HiFiAdapterFilt (Sim et al. 2022) and generated the initial phased diploid assembly using HiFiasm (Cheng et al. 2021) on Hi-C mode with the filtered PacBio HiFi reads and the Omni-C dataset. We then aligned the Omni-C data to both assemblies following the Arima Genomics Mapping Pipeline (https://github.com/ArimaGenomics/mapping_pipeline) and scaffolded both assemblies with SALSA (Ghurye et al. 2017, 2019).

**Table 1.** Assembly pipeline and software used.

	Software	Version
Assembly		
Filtering PacBio HiFi adapters	HiFiAdapterFilt https://github.com/sheinasim/HiFiAdapterFilt	Commit 64d1c7b
K-mer counting	Meryl	1
Estimation of genome size and heterozygosity	GenomeScope	2
De novo assembly (contigging)	HiFiasm	1.8
Long read, genome–genome alignment	Minimap2	2.16
Remove low-coverage, duplicated contigs	Purge_dups	1.0.1
Scaffolding		
Omni-C mapping for SALSA	Arima Genomics mapping pipeline https://github.com/ArimaGenomics/mapping_pipeline	Commit 2e74ea4
Omni-C scaffolding	SALSA	2
Gap closing	YAGCloser https://github.com/merlyescalona/yagcloser	Commit 0e34c3b
Omni-C contact map generation		
Short-read alignment	Bwa	0.7.17-r1188
SAM/BAM processing	Samtools	1.11
SAM/BAM filtering	pairtools	0.3.0
Pairs indexing	pairix	0.3.7
Matrix generation	Cooler	0.8.10
Matrix balancing	HiCExplorer	3.6
Contact map visualization	HiGlass	2.1.11
Benchmarking		
Basic assembly stats	QUAST	5.0.2
Assembly completeness	BUSCO	5.0.0
	Merqury	1

Software citations are listed in the text. SAM/BAM: Sequence alignment map format/ its compressed Binary format.

Both genome assemblies were manually curated by iteratively generating and analyzing their corresponding Omni-C contact maps. To generate the contact maps, we aligned the Omni-C data with BWA-MEM (Burrows-Wheeler Aligner Maximum Exact Match) (Li 2013), identified ligation junctions, and generated Omni-C pairs using pairtools (Open2C et al. 2024). We generated a multi-resolution Omni-C matrix with cooler (Abdennur and Mirny 2020) and balanced it with hicExplorer (Ramírez et al. 2018). We used HiGlass (Kerpedjiev et al. 2018) and the PretextSuite (https://github.com/wtsi-hpag/PretextView; https://github.com/wtsi-hpag/PretextMap; https://github.com/wtsi-hpag/PretextSnapshot) to visualize the contact maps, where we identified misassemblies and misjoins, and finally modified the assemblies using the Rapid Curation pipeline from the Wellcome Trust Sanger Institute, Genome Reference Informatics Team (https://gitlab.com/wtsi-grit/rapid-curation). Some of the remaining gaps (joins generated during scaffolding and curation) were closed using the PacBio HiFi reads and YAGCloser (https://github.com/merlyescalona/yagcloser). Finally, we checked for contamination using the BlobToolKit Framework (Challis et al. 2020).

### Genome quality assessment

We generated k-mer counts from the PacBio HiFi reads using meryl (https://github.com/marbl/meryl). The k-mer counts were then used in GenomeScope2.0 (Ranallo-Benavidez et al. 2020) to estimate genome features including genome size, heterozygosity, and repeat content. To obtain general contiguity metrics, we ran QUAST (Gurevich et al. 2013). To evaluate genome quality and functional completeness we used BUSCO (Manni et al. 2021) with the Actinopterygii ortholog database (actinopterygii_odb10), which contains 3,640 genes. Assessment of base level accuracy (quality value, QV) and k-mer completeness was performed using the previously generated meryl database and merqury (Rhie et al. 2020). We further estimated genome assembly accuracy via BUSCO gene set frameshift analysis using the pipeline described in Korlach et al. (2017). Measurements of the size of the phased blocks are based on the size of the contigs generated by HiFiasm on HiC mode. We follow the quality metric nomenclature established by Rhie et al. (2021), with the genome quality code *x*·*y*·*P*·*Q*·*C*, where *x* = log_10_[contig NG50]; *y* = log_10_[scaffold NG50]; *P* = log_10_[phased block NG50]; *Q* = Phred base accuracy QV; *C* = % genome represented by the first “n” scaffolds, following a karyotype of 2n = 44 estimated as the median number of chromosomes from other species in the same family (tax_tree [*E. newberryi*]; Genome on a Tree—GoaT, Challis et al. 2023). Quality metrics for the notation were calculated on the assembly for the primary haplotype.

### Genome assembly comparison within the Gobiidae

We searched on GenBank for all the Gobiidae genome assemblies available (query: https://www.ncbi.nlm.nih.gov/datasets/genome/?taxon=8220), downloaded all available genome assemblies, and selected a single genome assembly per species. Selection of the single genome assembly per species was made based on NCBI selection as “reference” or “representative genome.” We also examined and tabulated available exemplars from successive outgroups to Gobiidae in Gobiiformes. For all the genome assemblies, we generated contiguity metrics with QUAST and obtained metrics for functional completeness using BUSCO with the Actinopterygii ortholog database (actinopterygii_odb10).

## Results

**Fig. 2.**
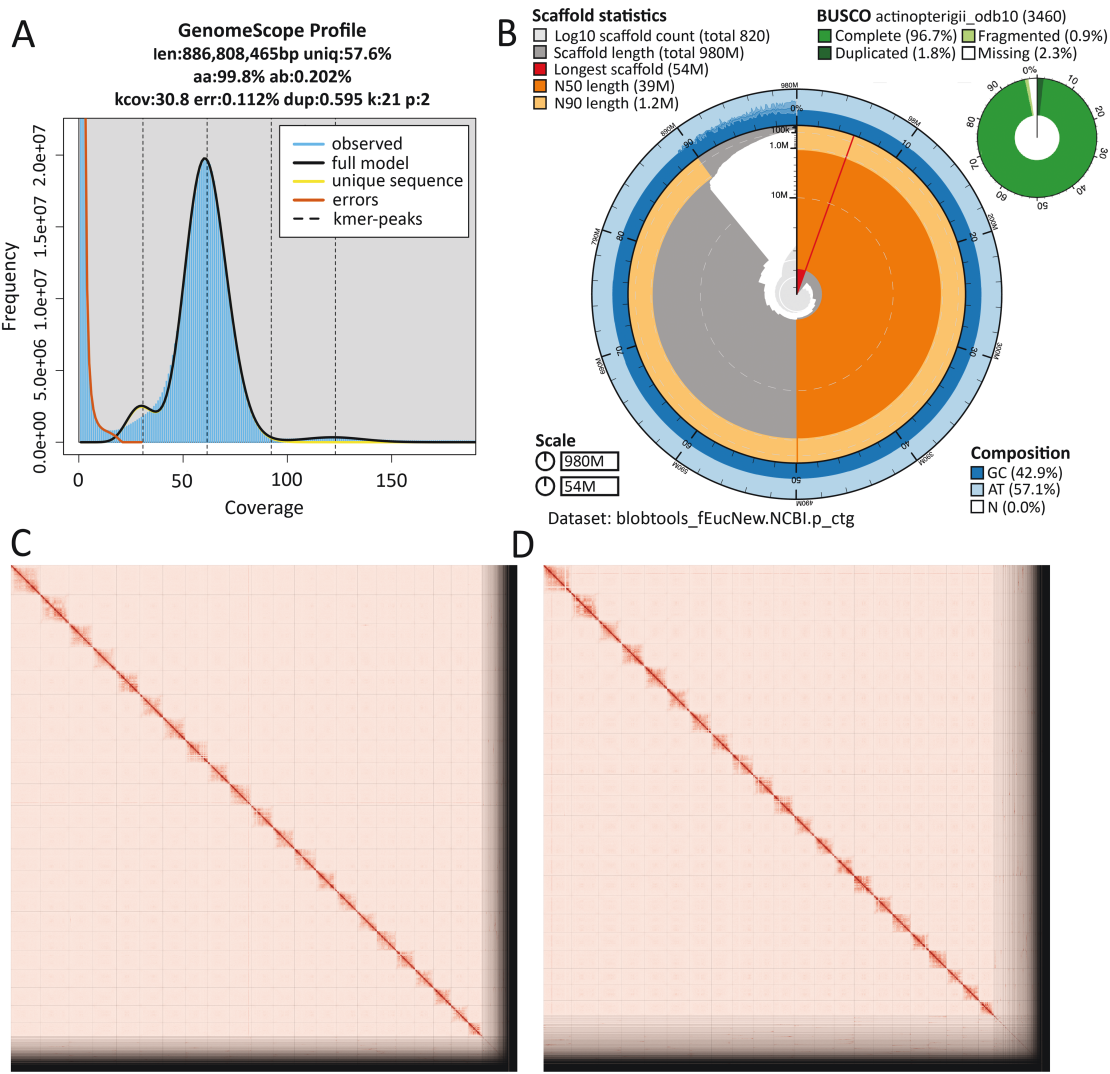
Visual overview of genome assembly metrics. A) K-mer spectrum output generated from PacBio HiFi data without adapters using GenomeScope2.0. The bimodal pattern observed corresponds to a diploid genome and the k-mer profile matches that of low (<1%) heterozygosity. K-mers covered at lower coverage and lower frequency correspond to differences between haplotypes, whereas the higher coverage and higher frequency k-mers correspond to the similarities between haplotypes. B) BlobToolKit Snail plot showing a graphical representation of the quality metrics presented in Table 2 for the *Eucyclogobius newberryi* primary assembly (fEucNew1). The plot circle represents the full size of the assembly. From the inside-out, the central plot covers length-related metrics in percent of the assembly. The thin radial line toward 1 o’clock represents the size of the longest scaffold; all other scaffolds are arranged in size order moving clockwise around the plot and drawn in gray starting from the outside of the central plot. Dark and light orange arcs show the scaffold N50 and scaffold N90 values 50 and 90 percent of the whole assembly. The central light gray spiral shows the cumulative scaffold count with a white line at each order of magnitude. White regions in this area reflect the proportion of Ns in the assembly; the dark versus light blue area around it shows mean, maximum, and minimum GC vs. AT content at 0.1% intervals (Challis et al. 2020). Omni-C contact maps for the primary (C) and alternate genome (D) assembly generated with PretextSnapshot. Omni-C contact maps translate the proximity of genomic regions in 3D space to contiguous linear organization. Each cell in the contact map corresponds to sequencing data supporting the linkage (or join) between two such regions.

The Omni-C and PacBio HiFi sequencing libraries generated 113.3 million read pairs and 5.71 million reads, respectively. The latter yielded ~63-fold coverage (N50 read length 10,125 bp; minimum read length 59 bp; mean read length 9,776 bp; maximum read length of 50,170 bp) based on the GenomeScope2.0 genome size estimation of 885.8 Mb. Based on PacBio HiFi reads, we estimated 0.112% sequencing error rate and 0.202% nucleotide heterozygosity rate. The limited bimodality of the k-mer spectrum (Fig. 2A) suggests minimal (<1%) heterozygosity consistent with the bottleneck history of the Topanga source population.

**Table 2.** Sequencing and assembly statistics, and accession numbers.

Bio-projects and vouchers	CCGP NCBI Bio-project	PRJNA720569 http://www.ncbi.nlm.nih.gov/bioproject/720569
	*Genus species* NCBI Bio-project	PRJNA896183 https://www.ncbi.nlm.nih.gov/bioproject/896183
	NCBI Bio-sample	SAMN31536049, SAMN31536050 https://www.ncbi.nlm.nih.gov/biosample/SAMN31536049 https://www.ncbi.nlm.nih.gov/biosample/SAMN31536050
	Specimen identification number	DKJ021-1_F, DKJ021-1_M
Genome sequence	PacBio HiFi long read runs (male/female)	1 PACBIO_SMRT (Sequel II) run: 5.7 M spots, 55.9 G bases
	Omni-C Illumina sequencing	2 Illumina NovaSeq 6000 run: 113.3 M spots, 34.2 G bases
	PacBio HiFi NCBI SRA Accession	SRR24455683 https://www.ncbi.nlm.nih.gov/sra/SRR24455683
	Omni-C Illumina NCBI SRA Accession	SRR24455684, SRR24455685 https://www.ncbi.nlm.nih.gov/sra/SRR24455684 https://www.ncbi.nlm.nih.gov/sra/SRR24455685
Genome assembly primary (alternate)	Assembly identifier	fEucNew1
	HiFi read coverage	63.10×
	Number of contigs	1,614 (1,649)
	Contig N50 (bp)	1,988,651 (1,700,290)
	Longest contigs	13,672,609 (12,786,001)
	Number of scaffolds	820 (755)
	Scaffold N50 (bp)	38,890,901 (39,256,509)
	Size of final assembly (bp)	984,795,555 (913,837,424)
	Gaps per Gbp	806 (978)
	NCBI Genome Assembly Accession	GCA_026437365.1 https://www.ncbi.nlm.nih.gov/assembly/GCA_026437365.1
Assembly quality	Assembly quality identifier	6.7.P.Q58.C
	Base pair QV (Merqury)	P: Q 58.2087, A: Q 58.1865
	Indel QV (frameshift analysis)	P: Q 47.79, A: Q 47.87
	k-mer completeness	P: 98.52%, A: 96.24%
	BUSCO completeness Primary (C:S:D:F:M) Alternate (C:S:D:F:M)	96.80%:95.00%:180%:0.90%:2.30% 95.20%:93.5%:1.70%:0.90%:3.90%
	Phased block NG50	2,315,297 bp (1,837,921 bp)

The final assembly (fEucNew1) consists of two phased haplotypes and both assemblies are slightly larger in size compared with the estimated value from GenomeScope2.0 (Fig. 2A; Pflug et al. 2020 ). Haplotype 1 (primary assembly) consists of 820 scaffolds spanning 984.7 Mb with contig N50 of 1.98 Mb, scaffold N50 of 38.8 Mb, longest contig of 13.6 Mb and largest scaffold of 54.29 Mb. Haplotype 2 (alternate) assembly consists of 755 scaffolds, spanning 913.74 Mb with contig N50 of 1.7 Mb, scaffold N50 of 39.25 Mb, largest contig of 12.78 Mb, and largest scaffold of 53.65 Mb (Table 2; Fig. 2B).

We manually generated a total of 45 breaks and 527 joins; 25 breaks were made on Haplotype 1 and 20 on the alternate Haplotype 2, and 325 joins were made on Haplotype 1 and 211 for Haplotype 2. This closed a total of 86 gaps, 41 on Haplotype 1 and 45 on Haplotype 2. Finally, we filtered out a single contig from Haplotype 1 corresponding to Mollusca contamination. No other contigs were removed.

Haplotype 1 has a BUSCO completeness score of 96.8% using the Actinopterygii gene set, a per base quality (QV) of 58.20, a k-mer completeness of 98.52%, and a frameshift indel QV of 47.79. Haplotype 2 has a BUSCO completeness score of 95.2% using the same gene set, a per base quality (QV) of 58.18, a k-mer completeness of 98.52%, and a frameshift indel QV of 47.87. The Omni-C contact maps show that both assemblies are highly contiguous, with some chromosome-length scaffolds. The contact maps also show that the assemblies resolve the data to 22 contigs comprising 88% of the data (Fig. 2C and D), matching the expected karyotype for the species. We have deposited both assemblies on NCBI (Table 2).

## Discussion

We briefly compare the NTG genome to other goby genomes (see Supplementary Table S1 and its caption for phylogenetic discussion), then discuss the particulars of our genome. The 0.980 Gb estimated genome size of NTG falls comfortably within the expected range of genome sizes for related gobies. The blind goby (*Typhlogobius californiensis*), the closest NTG relative considered, has a genome size estimate of 1.20 Gb (Ellingson et al. 2014) based on k-mer estimations from short reads. Other goby genome sizes determined via seqeuencing (Supplementary Table S1) are considered as a succession of outgroups (McCraney et al. 2020). *Chaenogobius annularis* (0.747 Gb) falls within the North Pacific bay goby group, which also contains *Eucyclogobius* (Ellingson et al. 2014). *Rhinogobius similis* (0.890 Gb) and *Mugilogobius chulae* (1.000 Gb) fall within the broader Gobionellinae and have similarly small genomes. The succeeding outgroup includes the eel gobies (Amblyopinae) and the mudskippers (Oxudercinae), where nine genomes ranging in size from 0.753 to 1.193 Gb have been sequenced. Six genomes have been sequenced in the outgroup to these, Benthophilinae + Gobiinae): the five genomes from within the Gobiinae appear consistently small ranging from 0.563 to 0.873 Gb. Beyond the Gobiidae as applied here (see Supplementary Table S1), successive outgroups among the “sleeper” gobies Butidae, Eleotridae, Odontibutidae, and Rhyacichthyidae are comparable in size. The last outgroup considered, Apogonidae (cardinalfishes), may show a slight increase in genome size relative to the ingroup genome size. With genome sequence in hand, further genomic comparisons are merited beyond simple metrics of genome size (e.g. Gregory 2023). With a few exceptions, these genomes show BUSCO scores over 90% and appear to be of high quality.

Genome sequencing effort within the gobies does not correlate with subgroup species diversity as quantified by Fricke et al. (2024). The mudskippers and eelgobies with a total species diversity of 82 have 9 genomes sequenced due to the strong research interest in terrestrialization in mudskippers (e.g. Li et al. 2022), while the round goby, a lacustrine benthic specialist, was sequenced in part due its invasion of the North American Great Lakes (Adrian-Kalchhauser et al. 2020). The Gobionellinae, the subfamily to which NTG belongs with a diversity of 523 species, now has 4 sequenced species (e.g. Cai et al. 2021), and we hope that this will help develop this group and the North Pacific bay gobies within it as objects of further research. It is also noteworthy that the subfamily Gobiinae with 1,313 species has only 5 sequenced genomes which appear to be of consistently small size (Supplementary Table S1) and may deserve further reference genome development, as may several lower diversity groups with the broader Gobiiformes which have no high-quality reference genome.

Approximately 88% of the NTG genome is mapped to 22 contigs, identical to the chromosome number found in the yellowstripe goby (Cai et al. 2021), and similar to the 24 chromosomes determined using sequencing in the walking goby (Li et al. 2022). In the standard genome size database, the chromosome number among 13 species of Gobiidae ranges from 20 to 25, with a mean of 23 chromosomes (Gregory 2023).

Although locally isolated in lagoons and endemic to California, the genus *Eucyclogobius* occurs from the northernmost (Del Norte) to the southernmost (San Diego) California coastal counties.

These short-lived, typically annual fishes experience seasonal population fluctuations where evolution can occur over very short timescales. In addition, multiple lagoons that are subject to changing hydrology, temperature, and disease impacts (Jacobs et al. 2011; Spies and Steele 2016; Feyrer et al. 2023) suggesting parallel or replicate adaptive evolution (Ahnelt et al. 2004; Chase et al. 2016; Swift et al. 2016). In addition, drought/flood cycles expose management subunits to local extinction/recolonization metapopulation dynamics (Lafferty et al. 1999; Kinziger et al. 2015; Martel et al. 2021). These processes, as well as the specific history of the genome source population from Topanga Lagoon, a recently recolonized population, are consistent with the low heterozygosity observed (Manion 1993; Spies 2022). Given these population and species-level attributes, this genome provides an important resource for scientific study and conservation management for both the NTG and the highly bottlenecked populations of STG (Dawson et al. 2001; Earl et al. 2010; Swift et al. 2016).

More broadly, tidewater gobies provide a prime study system for evolutionary investigation given their propensity for bottleneck, genetic drift, and parallel natural selection in isolated populations, as well as their variable metapopulation dynamics. TWG habitats are strongly affected by hydrology and climatic variables. Precipitation events open their lagoon habitats to the sea permitting local dispersal, while temperature and desiccation are often associated with extirpation. Consequently, tidewater goby demographics, selection, and metapopulation process are all strongly influenced by climate. An increase in hydrologic variation with anthropogenic climate change should substantially influence these fishes. Consequently, our new reference genome for *Eucyclogobius* is timely and will serve as a foundation for evolutionary population genomics studies, contributing to the conservation and management efforts for these endangered taxa.

## Supplementary material

Supplementary material is available at *Journal of Heredity* online.

esae053_suppl_Supplementary_Material

## Data Availability

Data generated for this study are available under NCBI BioProject PRJNA720569. Raw sequencing data for samples DKJ021-1_F (fin), DKJ021-1_M (muscle) (NCBI BioSamples SAMN31536049 and SAMN3153605) are deposited in the NCBI Short Read Archive (SRA) under SRR24455683, SRR24455684, and SRR24455685. Assembly scripts and other data for the analyses presented can be found at the following GitHub repository: www.github.com/ccgproject/ccgp_assembly.
