# Supplementary material for: Reference genome for the endangered, genetically subdivided, northern tidewater goby, Eucyclogobius newberryi
Source: J Hered. 2024 Oct 5;116(2):170–8. doi: 10.1093/jhered/esae053 (PMC11879183; doi:10.1093/jhered/esae053)
Supplement: esae053_suppl_Supplementary_Material [file esae053_suppl_supplementary_material.docx]

**
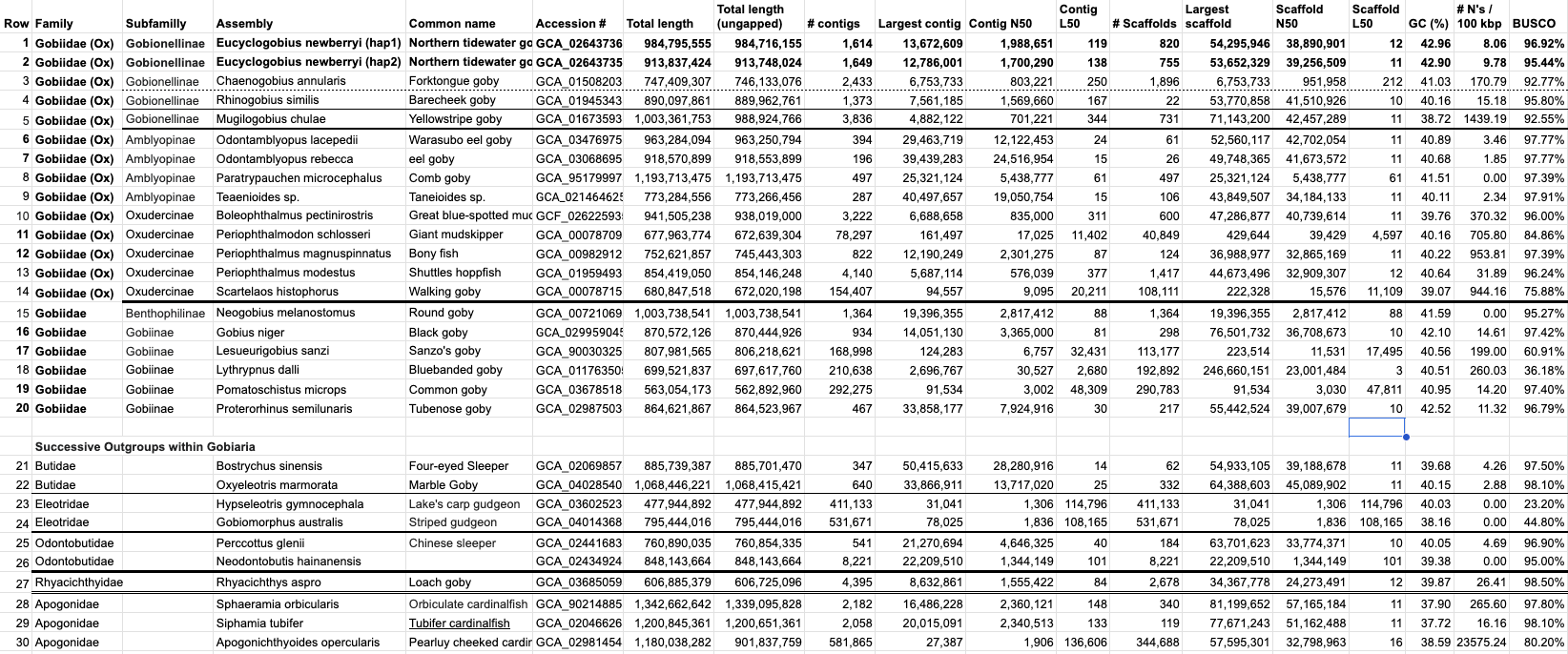
**

**Supplemental Table 1 Caption- Goby genome comparison to the NTG genome**

This table provides standard comparative metrics for genomes of gobies and allied taxa listed relative to their phylogenetic proximity to the northern tidewater goby (NTG) genome reported here. It should be noted that goby taxonomy is in flux and that aspects of basal topology have been difficult to resolve (e.g. McCraney et al. 2020). Consequently informal phylogenetic terms have often been used. Recent authoritative treatments (e.g. Near and Thacker 2024; Van der Laan et al. 2024) divide the “Gobiidae'' taxon used in Genbank, the basis of this table, into two families: Oxudercidae and Gobiidae. Furthermore there appears to be no formal taxonomic name for the taxon including both of the now sister families Oxudercidae and Gobiidae, although Gierl et al (2022) refers to this group as 5brG based on their synapomorphic reduction of branchiostegal rays to 5. To accommodate this situation, “Gobiidae (Ox)” in the family column of the table represent those genomes now placed in Oxudercidae, while “Gobiidae” is used for genomes from taxa that have been retained in that family. Only those families and subfamilies with genomic representation are listed in the table. The successive larger phylogenetic groupings designated by rows include 1-3 North Pacific bay gobies (e.g. Ellingson et al. 2014), 1-4 Acanthogobius group (e.g. McCraney et al. 2020), 1-5 Gobionellinae (523 species), 1-14 Oxudercidae [Gobiidae (Ox)] (725 species), 1-22 traditional gobies/5brG (Oxudercidae + Gobiidae) 2141 species, and 15-20 Gobiidae (1416 species). Successive outgroups to traditional gobies include 20-21 Butidae (55 species), 22-23 Eleotridae (152 species), 24-25 Odontobutidae (26 species), 26 Rhyacichthyidae (4 Species), and 27-30 Apogonidae (381 species). Species numbers are after Fricke et al. (2024). Butidae does not fall within Eleotridae (e.g. McCraney et al. 2020), so it is treated as a family.

Reference:

Gierl C, Dohrmann M, Keith P, Humphreys W, Esmaeili HR, Vukić J, Šanda R, Reichenbacher B. An integrative phylogenetic approach for inferring relationships of fossil Gobioids (Teleostei: Gobiiformes). *PLoS One*. 2022:17:e0271121. <https://journals.plos.org/plosone/article?id=10.1371/journal.pone.0271121>

Near TJ, Thacker CE. Phylogenetic classification of living and fossil ray-finned fishes (Actinopterygii). *Bulletin of the Peabody Museum of Natural History* 2024:65(1):3–02.

Van der Laan R, Fricke R, Eschmeyer WN, editors. Eschmeyer’s Catalog of Fishes: classification. 2024 [accessed 2024 Jul 31]. <http://www.calacademy.org/scientists/catalog-of-fishes-classification/>
